# Supplementary figures and images for: A seven-sex species recognizes self and non-self mating-type via a novel protein complex
Source: eLife. 2024 Feb 28;13:RP93770. doi: 10.7554/eLife.93770 (PMC10901506; doi:10.7554/eLife.93770)

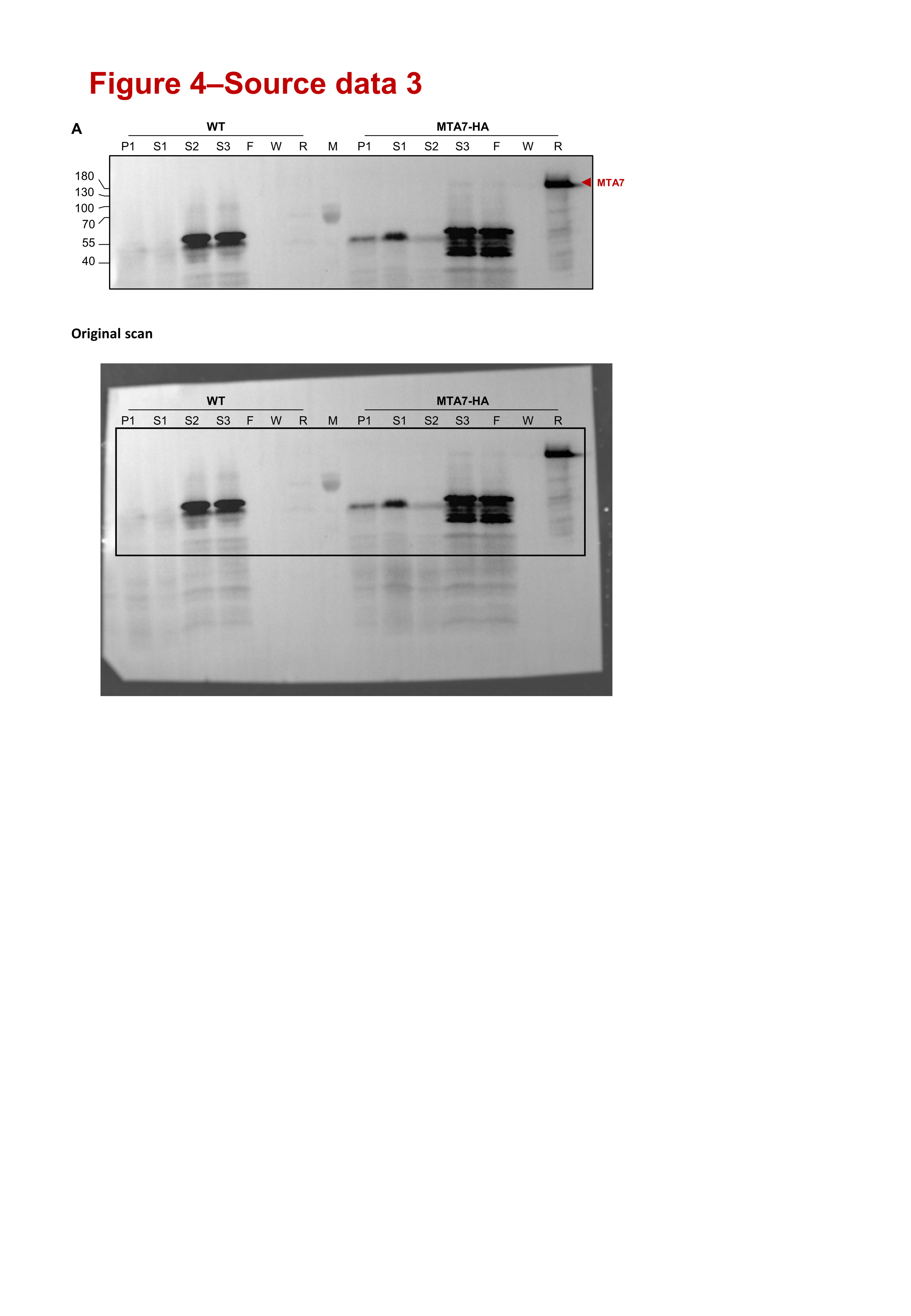

Supplement: Figure 4—source data 3. [file elife-93770-fig4-data3.zip › Figure 4-Source data 3.tif]

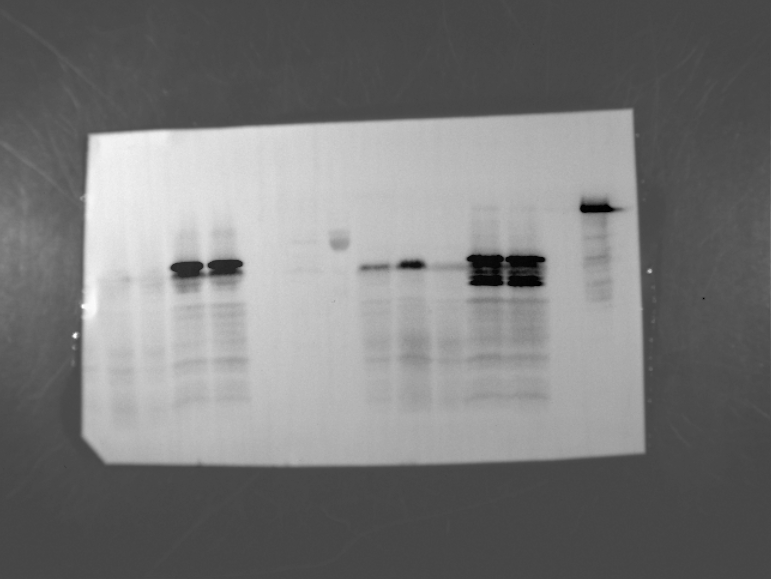

Supplement: Figure 4—source data 3. [file elife-93770-fig4-data3.zip › Figure 4-Source data 3-raw blot.tif]

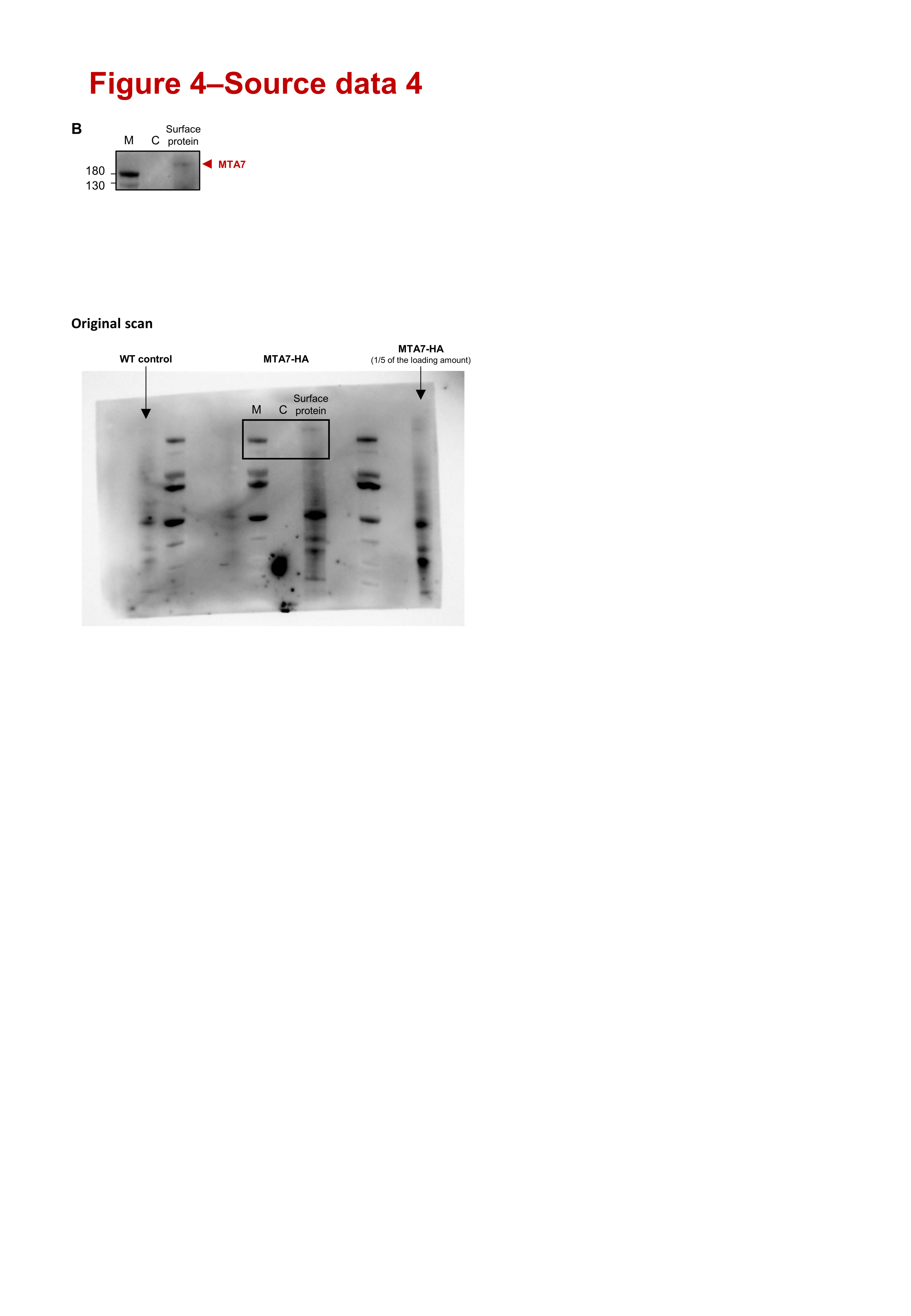

Supplement: Figure 4—source data 4. [file elife-93770-fig4-data4.zip › Figure 4-Source data 4.tif]

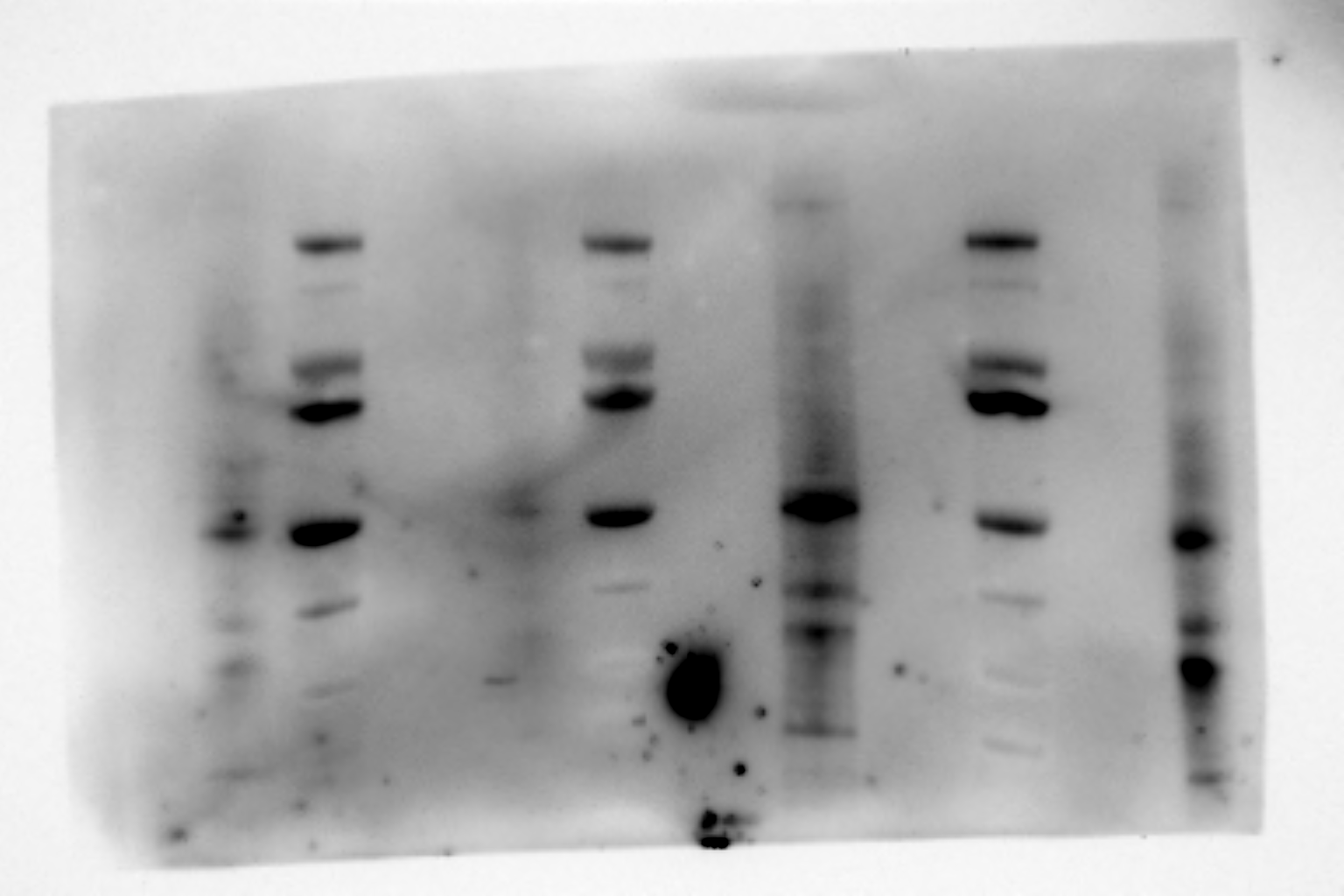

Supplement: Figure 4—source data 4. [file elife-93770-fig4-data4.zip › Figure 4-Source data 4-raw blot.bmp]

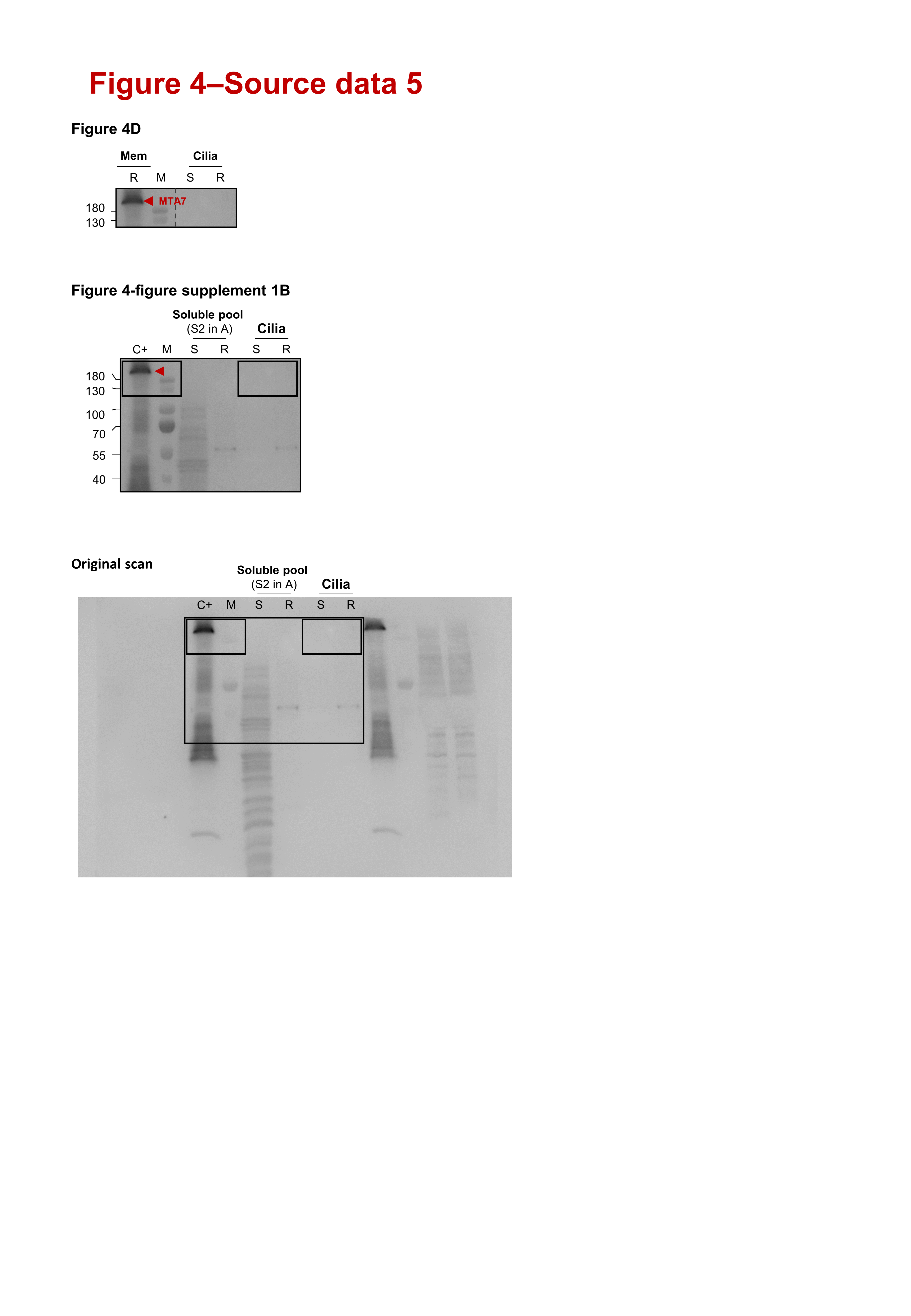

Supplement: Figure 4—source data 5. [file elife-93770-fig4-data5.zip › Figure 4-Source data 5.tif]

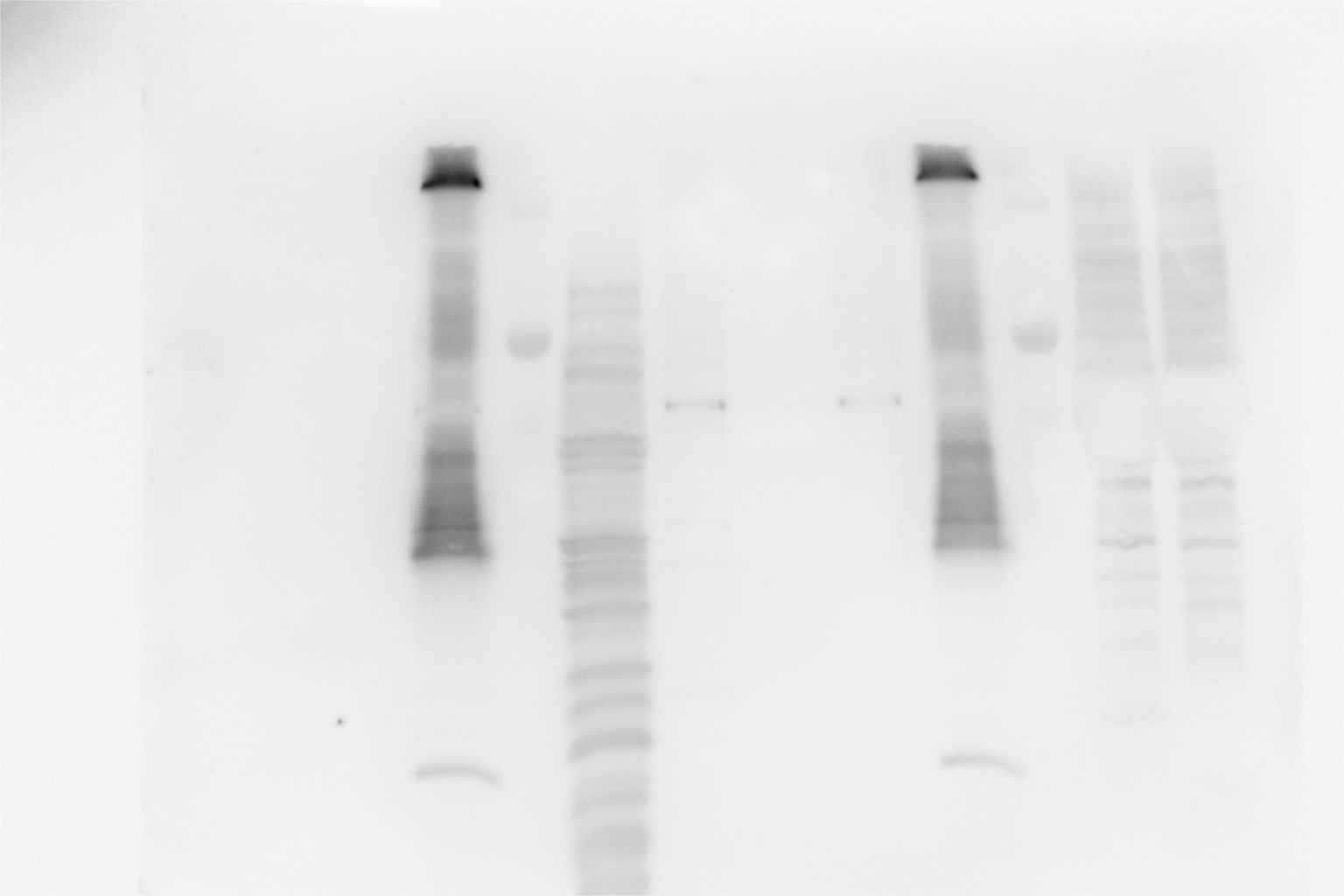

Supplement: Figure 4—source data 5. [file elife-93770-fig4-data5.zip › Figure 4-Source data 5-raw blot.png]
